# Supporting Non-Psychiatric Trainees to Engage with Reflective Practice and Attend to Their Wellbeing Through Balint Group

**DOI:** 10.1192/bjo.2025.10366

**Published:** 2025-06-20

**Authors:** Ayatalla Gabre, Elinor Bradley

**Affiliations:** Kent and Medway NHS and Social Care Partnership Trust, Canterbury, United Kingdom

## Abstract

**Aims:** Doctors in training report high rates of burnout. The Balint group lends itself to addressing emotional stress and hence the associated risk of burnout. However, Balint group attendance among GP trainees and foundation doctors locally has been poor compared with psychiatric trainees. A Quality Improvement project was undertaken to explore and address barriers to attendance with the aim of improving GP trainees’ and foundation doctors’ engagement with the Balint group.

**Methods:** QI methodology was used throughout 2024. We implemented a quantitative, cross-sectional design using anonymous online surveys. We used purposive sampling by sending the surveys to GP trainees and foundation doctors on psychiatric placements within Kent and Medway NHS and Social Care Partnership Trust (KMPT). The survey was semi-structured, with closed and open-ended responses. The survey explored their understanding of the Balint group, how important they perceived it to be, and the barriers they experienced to attending.

Data gathered informed several ‘change ideas’ which were implemented through consecutive plan-do-study-act (PDSA) cycles. The timing of Balint groups was changed to ensure that less-than-full-time doctors had options to attend and that groups were less likely to conflict with clinical commitments. Improvements were made to the induction process to better socialise non-psychiatric trainees with the Balint group. A face-to-face format was trialled, replacing the previous virtual format.

Post-intervention surveys were administered, which included validated measures of burnout (Abbreviated Copenhagen Burnout Inventory).

**Results:** Resident doctors’ understanding of the Balint group’s function and process has improved. In parallel, attendance has increased in some Balint groups; for example, 75% attendance in June 2024 compared with 25% in March 2024. However, with frequent rotations of GP trainees and foundation doctors, each cohort having its own needs and preferences, we have found that improvements are not consistently sustained. Barriers still exist, such as conflicts with clinical commitments and the format feeling ‘alien’ and unhelpful to others. Changes to the degree of burnout through attending the Balint group are inconclusive and will be clarified with the results of a follow-up survey in March 2025.

**Conclusion:** GP trainees and foundation doctors are better able to engage with the Balint group when barriers to attendance are actively addressed. However, not all resident doctors feel comfortable with the Balint group format, and hence it may not reduce the risk of burnout for these individuals; in such cases, attendance should not be mandated.

